# Comparative Analysis of De Novo Immune Thrombocytopenia Following mRNA COVID-19 Vaccine Versus Non-mRNA Vaccines and COVID-19: A Global Database Analysis

**DOI:** 10.7759/cureus.41460

**Published:** 2023-07-06

**Authors:** Meric Mericliler

**Affiliations:** 1 Hematology, Medical Oncology, and Palliative Care, Virginia Commonwealth University, Richmond, USA; 2 Hematology, Medical Oncology, and Palliative Care, Massey Cancer Center, Richmond, USA

**Keywords:** hematologic autoimmune disorders, vaccine, vaccine hesitancy, mrna-based vaccine, auto immune, immune thrombocytopenia (itp), covid 19

## Abstract

Introduction

Autoimmune diseases have been linked to COVID-19 vaccines. An increasing number of cases have reported de novo immune thrombocytopenia (ITP) following mRNA COVID-19 vaccines. This study aims to investigate the incidence of de novo ITP following the mRNA COVID-19 vaccine in comparison to other non-mRNA vaccines and COVID-19.

Methods

Data were collected from the TriNetX global health research network, which covers over 117 million patients. Four different patient cohorts were included: those who received the mRNA COVID-19 vaccine (between 12/15/2020 - 5/1/2023), the influenza vaccine (between 01/01/2010 - 01/01/2020), tetanus, diphtheria, and pertussis/tetanus and diphtheria (Tdap/Td) vaccines (between 01/01/2010 - 01/01/2020), and those who had COVID-19 (between 01/01/2020 - 05/01/2023). A comparative analysis was conducted to examine the occurrence of de novo ITP within three weeks after receiving mRNA COVID-19 vaccine, non-mRNA vaccines, or upon diagnosis of COVID-19. Additionally, a comparative analysis was performed after 1:1 propensity score matching to balance baseline characteristics (age, sex, and race).

Results

The overall event rate was 0.07 per 10,000 for the mRNA COVID-19 vaccine, 0.25 per 10,000 for the influenza vaccine, and 0.28 per 10,000 for the Tdap/Td vaccines. Additionally, the incidence of de novo ITP following COVID-19 was 0.30 per 10,000. Those who received the influenza vaccine and Tdap/Td vaccines had higher rates of de novo ITP compared to the mRNA COVID-19 vaccine group, with a relative risk of 3.48 and 3.88, respectively. The occurrence of de novo ITP following COVID-19 was significantly higher compared to that following the mRNA COVID-19 vaccine, with a relative risk of 4.27. Post-propensity score matching analysis produced similar outcomes.

Conclusions

The findings of this study suggest that the incidence of de novo ITP is significantly lower following mRNA-based COVID-19 vaccines compared to non-mRNA vaccines and COVID-19.

## Introduction

The coronavirus disease 2019 (COVID-19) pandemic has instigated an unprecedented rush toward developing and distributing effective vaccines. Messenger RNA (mRNA) vaccines for COVID-19 have proven to be highly effective in randomized trials, substantially reducing the risk of COVID-19, especially severe disease, and have been the most widely administered vaccines against COVID-19 [1,2]. Nevertheless, concerns have been raised about their safety and potential adverse effects, including autoimmune disorders [3,4]. An increase in autoimmune diseases has been associated with antigenic cross-reactivity between severe acute respiratory syndrome coronavirus 2 (SARS-CoV-2) and human tissue [5].

Among the autoimmune diseases reported following mRNA COVID-19 vaccinations, hematologic autoimmune conditions, particularly de novo immune thrombocytopenia (ITP), have gained increased attention due to a rising number of reports [4,6,7]. In the majority of these cases, ITP occurred after the first dose of the vaccine and displayed a shorter lag time compared to ITP following routine childhood vaccines. A recent study utilizing the US Centers for Disease Control's (CDC) Vaccine Adverse Event Reporting System (VAERS) database, however, indicated that autoimmune hematologic diseases are rare after immunization for COVID-19, with rates appearing to be lower than those in the general population [8]. Yet, no studies have directly compared the incidence of ITP following COVID-19 vaccinations to that after other vaccines or COVID-19.

This study aims to investigate the incidence of de novo ITP following administration of mRNA COVID vaccine, and to provide a comparative analysis with the incidence following non-mRNA vaccines and COVID-19 infections, utilizing a comprehensive global database.

## Materials and methods

Retrospective de-identified patient data were collected from the TriNetX database (Cambridge, USA), a global federated database that covers over 117 million patients. The TriNetX database has previously been described in the literature [9]. This study included four distinct patient cohorts:

1. Patients who received the mRNA COVID-19 vaccine from December 15, 2020, to May 1, 2023.

2. Patients diagnosed with COVID-19 from January 1, 2020, to May 1, 2023.

3. Patients who received the influenza vaccine from January 1, 2010, to January 1, 2020.

4. Patients who received the Tdap/Td (tetanus, diphtheria, and pertussis/tetanus and diphtheria) vaccines from January 1, 2010, to January 1, 2020.

The dates for the COVID‐19 vaccine and COVID-19 groups range from the first day of mRNA-based vaccine administration and the first COVID-19 diagnosis worldwide, respectively [10]. The other three vaccinated groups were examined between January 1, 2010, to January 1, 2020, to exclude the possibility of SARS-CoV-2 infection or COVID‐19 vaccination in these cohorts. 

The study included only adult patients (age ≥18 years). Additionally, patients were included only if they had at least one healthcare visit prior to the index event. Patients with a history of ITP before the index event were excluded. Those who received other vaccinations or had COVID-19 (except for the COVID-19 group) within three weeks of the index event were also excluded.

De novo ITP was defined as an encounter with a diagnosis of ITP in patients with no prior history of ITP. Patient cohorts and outcomes were identified using relevant diagnostic codes, as described in the supplementary data (Appendix A). The occurrences of de novo ITP following mRNA COVID-19 vaccination, non-mRNA vaccinations, and SARS-CoV-2 infection within three weeks were examined and compared. The three-week timeline was selected based on previous reports indicating that most de novo cases occur within three weeks following the COVID-19 vaccines [7].

The study was exempt from institutional review board approval as it utilized de-identified patient records on the TriNetX platform. Descriptive statistics were presented as means and standard deviations or percentages. Risk ratios (RR) with 95% confidence intervals (CI) were calculated for each outcome. A 1:1 propensity score matching (PSM) procedure was performed using TriNetX's logistic regression model to balance baseline characteristics including age, sex, and race. Among the races, only white and black individuals were included in the PSM analysis due to the limitation in the number of variables available for the analysis. Outcome analysis was conducted before and after propensity score matching. All statistical analyses were performed on the TriNetX platform.

## Results

Baseline characteristics varied across the groups, with the mRNA COVID-19 vaccine group having a higher mean age, and fewer Black patients (Table 1). After 1:1 propensity score matching, the groups were balanced in terms of demographics (Appendix B, C, & D).

**Table 1:** Demographic characteristics of the patient groups. Tdap/Td denotes tetanus, diphtheria, and pertussis/tetanus and diphtheria.

	mRNA COVID‐19 vaccine	COVID-19	Influenza vaccine	Tdap/Td vaccines
	(n = 3,177,285)	(n = 12,541,581)	(n = 2,355,057)	(n = 1,817,211)
Age (mean, SD)	52.2 ± 19.1	48.4 ± 19.4	47.2 ± 21.7	41.4 ± 18.9
Female sex (%)	57%	56%	59%	56%
Race				
White (%)	63%	51%	70%	67%
Black (%)	11%	13%	13%	15%

The overall event rate was 0.07 per 10,000 for the mRNA COVID-19 vaccine, 0.25 per 10,000 for the influenza vaccine, and 0.28 per 10,000 for the Tdap vaccine. Additionally, the incidence of de novo ITP following COVID-19 was 0.30 per 10,000. 

Patients who were diagnosed with COVID-19 had a significantly higher incidence of de novo ITP within 21 days compared to those who received the mRNA COVID-19 vaccine. The relative risk prior to propensity score matching (PSM) was 4.27 (95% CI: 2.83 - 6.45). After balancing baseline characteristics, the relative risk was 4.96 (95% CI: 3.13 - 7.83).

For recipients of the influenza vaccine, the relative risk of de novo ITP was 3.48 (95% CI: 2.17 - 5.59), compared to those who received the mRNA COVID-19 vaccine. After applying PSM, the relative risk for de novo ITP increased to 4.83 (95% CI: 2.59 - 8.99). Lastly, patients who received Tdap/Td vaccines had a relative risk of 3.88 (95% CI: 2.39 - 6.31) compared to the mRNA COVID-19 vaccine before PSM, and a relative risk of 4.8 (95% CI: 2.43 - 9.49) after PSM.

Detailed event rates and comparative data are provided in Table 2.

**Table 2:** Comparison of the risk for a new diagnosis of ITP among different patient groups. Data are presented as No. (%) unless otherwise indicated. PSM denotes propensity score matching, and ITP denotes immune thrombocytopenia. Tdap/Td denotes tetanus, diphtheria, and pertussis/tetanus and diphtheria.

	Number of patients	Number of patients with de novo ITP	Relative risk for de novo ITP compared to mRNA COVID‐19 vaccine (before PSM)	Relative risk for de novo ITP compared to mRNA COVID‐19 vaccine (after PSM)
mRNA COVID‐19 vaccine	3,364,656	24 (0.001%)	1	1
COVID-19	12,772,798	389 (0.003%)	4.27 (2.83 - 6.45)	4.96 (3.13 - 7.83)
Influenza vaccine	2,375,072	59 (0.002%)	3.48 (2.17 - 5.59)	4.83 (2.59 - 8.99)
Tdap/Td vaccines	1,841,019	51 (0.003%)	3.88 (2.39 - 6.31)	4.8 (2.43 - 9.49)

## Discussion

With the emergence of the COVID-19 pandemic, the spotlight has turned to the speed of vaccine development, particularly that of mRNA vaccines. These vaccines have demonstrated high efficacy rates in large, randomized trials [1,2]. However, their expedited development has also raised concerns regarding safety and potential side effects, including autoimmune disorders [3,4]. The exact mechanism of vaccine-induced autoimmunity remains elusive, although molecular mimicry and immune cross-reactivity are implicated in its pathophysiology [5,11]. Among the SARS-CoV-2 antigens, the spike glycoprotein has been implicated in the pathogenesis of autoimmune diseases through immune cross-reactivity [12]. There has been a noteworthy increase in the reported cases of hematologic autoimmune conditions, including de novo ITP, following the administration of mRNA-based COVID-19 vaccines [7]. 

A recent systematic review included a total of 77 patients with de novo COVID-19 vaccine-associated ITP reported in the literature. Most cases of ITP developed after mRNA-based vaccinations, specifically after the first dose. Notably, 75% of these patients developed ITP within 12 days of vaccination, indicating a short lag time [7]. Mesina et al. conducted a study investigating hematologic adverse events after COVID-19 vaccination in the Philippines, using a national database [13]. They reported a low incidence rate of ITP, with an event rate of 0.0007 per 10,000 vaccine doses. A similar study from the United States, using the CDC's VAERS, also indicated a low incidence of hematologic events (1.05 per 1,000,000 doses) following a bivalent COVID-19 booster vaccination [8]. The authors further demonstrated the rarity of ITP following vaccinations, showing a variance in incidence among the three vaccine manufacturers (0.039 cases per 100,000 doses of Pfizer-BioNTech, 0.055 cases per 100,000 doses of Moderna, and 0.133 cases per 100,000 doses of J&J/Janssen). Importantly, most reported cases cannot definitively be attributed to the vaccination, as distinguishing vaccine-induced ITP from coincidental ITP can be challenging [14]. 

Consistent with the literature, this study using real-world data also shows a low incidence of ITP following mRNA-based COVID-19 vaccinations, with an event rate of 0.07 per 10,000 vaccines, albeit higher than the reported data. This is the first study comparing the occurrences of ITP after mRNA COVID-19 vaccines and non-mRNA vaccines, showing a significantly lower rate of ITP following mRNA COVID-19 vaccines compared to other vaccines.

Thrombocytopenia can also occur in the context of SARS-CoV-2 infection due to various mechanisms. Immune thrombocytopenia has been reported as a complication of COVID-19, potentially due to factors such as molecular mimicry, immune dysregulation, cryptic antigen expression on platelets, and epitope spreading [15-17]. No studies thus far have directly compared the incidence of ITP following SARS-CoV-2 infection and mRNA COVID-19 vaccination. The current study reveals a significantly increased incidence of ITP following SARS-CoV-2 infection compared to mRNA COVID-19 vaccination.

There were several limitations to the current study. It was designed retrospectively, utilizing a large dataset, both factors that inherently lead to potential selection bias and incomplete data. For instance, this study might not have captured less severe ITP cases, especially those not seeking medical attention, in the analysis. Another limitation is the reliance on diagnostic and procedure codes for data extraction, given the possibility of inaccuracies or errors in coding. ITP is generally a diagnosis of exclusion, which raises the possibility that the number of cases identified as ITP may be overestimated. Moreover, it is acknowledged that although rare, patients who were vaccinated against COVID-19 might have had a COVID-19 infection within three weeks following vaccination (or vice versa), and these patients are included in the analysis.

It is worth noting that this study did not provide a comparative analysis for de novo ITP following mRNA-based COVID-19 vaccination versus non-mRNA COVID-19 vaccination (e.g., adenovirus-based vaccines) due to the following reasons. First, adenovirus-based vaccines constitute less than 3% of the vaccines administered in the United States, and only a limited number of patients who received non-mRNA COVID-19 vaccines were available for analysis [18]. Second, vaccine‐induced immune thrombotic thrombocytopenia (VITT) has been reported among patients receiving adenoviral vectored COVID-19 vaccines, and these cases might be labeled as ITP since VITT does not have a specific diagnostic code.

Only patients with a new diagnosis of ITP were included in the analysis; however, this could potentially overlook patients with previously diagnosed ITP who were not registered in the database. To avoid this, only those who had at least one healthcare visit prior to the index event were included in this study. The three-week timeline was selected based on previous reports indicating that most de novo cases occur within three weeks following the COVID-19 vaccines [7]. De novo ITP cases can also occur later than three weeks after vaccination. However, a longer time window from the index event to the outcome, leading to a weaker temporal relationship, would potentially decrease the strength of the causal relationship. It's also noteworthy that several studies have indicated that certain demographics may be at a higher risk for vaccine-related ITP [8]. For this reason, a 1:1 propensity score-matching was performed to balance baseline characteristics, and outcomes were similar in the post-PSM analysis. 

## Conclusions

In conclusion, this study suggests a significantly lower rate of de novo immune thrombocytopenia following mRNA COVID-19 vaccination compared to rates found in patients receiving non-mRNA vaccines or those diagnosed with COVID-19. While the incidence of ITP post mRNA COVID-19 vaccination is low, it is higher compared to previous reports. These findings contribute vital evidence to the ongoing discourse on the safety profile of mRNA COVID-19 vaccines. Given the global scale of the COVID-19 vaccination program, continuous monitoring and vigilant reporting of potential side effects remain of utmost importance.

## Appendices

Appendix A 

**Table 3:** List of procedural and diagnostic codes used for obtaining data from the database. *Indicates an International Classification of Diseases, Tenth Revision, Clinical Modification (ICD-10-CM) diagnostic code. ITP denotes immune thrombocytopenia, Tdap/Td denotes tetanus, diphtheria, and pertussis/tetanus and diphtheria.

Vaccine/condition	Associated codes		
mRNA COVID-19 vaccine	2468231, 91312, 91300, 91305, 91301, 0002A, 91314, 0011A, 0012A, 0001A, 91313, 91311, 91315, 91309, 91306, 91307, 91308, 90656, 90658, 90686, 90688, 90662, 90714, 90715		
COVID-19	U07.1*, U07.2*, 94500-6, 94534-5, 94558-4, 94316-7, 94559-2, 94759-8, 96119-3, 94565-9, 94309-2, 94763-0, 94760-6, 94845-5, 96763-8, 94533-7, 94314-2, 95406-5, 96123-5, 94757-2, 94308-4, 94307-6, 95409-9, 97097-0, 9088, 95608-6		
Influenza vaccine	90662, 90656, 90688, 90686, 90685, 90658											
Tdap/Td vaccines	90715, 90714		
ITP	D69.3*		

Appendix B 

**Table 4:** Patient number and demographic characteristics in cohort 1 (COVID-19) and cohort 2 (mRNA vaccine) before and after propensity score matching.

Cohort 1 and cohort 2 patient count before and after propensity score matching											
			Cohort			Patient count before matching			Patient count after matching		
			1 - COVID-19			12,541,581			3,177,285		
			2 - mRNA vaccine			3,177,285			3,177,285		
Cohort 1 (N = 12,541,581) and cohort 2 (N = 3,177,285) characteristics before propensity score matching											
	Demographics										
		Cohort				Mean ± SD	Patients	% of Cohort		P-Value	Std diff.
		1 2		AI	Age at Index	48.4 +/- 19.4 52.2 +/- 19.1	12,541,581 3,177,285	100% 100%		<0.001	0.196
		1 2		2106-3	White		6,351,334 2,011,825	50.6% 63.3%		<0.001	0.258
		1 2		F	Female		7,055,860 1,801,876	56.3% 56.7%		<0.001	0.009
		1 2		2054-5	Black or African American		1,672,547 362,803	13.3% 11.4%		<0.001	0.058
Cohort 1 (N = 3,177,285) and cohort 2 (N = 3,177,285) characteristics after propensity score matching											
	Demographics										
		Cohort				Mean ± SD	Patients	% of Cohort		P-Value	Std diff.
		1 2		AI	Age at Index	52.2 +/- 19.1 52.2 +/- 19.1	3,177,285 3,177,285	100% 100%		1	<0.001
		1 2		2106-3	White		2,011,825 2,011,825	63.3% 63.3%		1	<0.001
		1 2		F	Female		1,801,876 1,801,876	56.7% 56.7%		1	<0.001
		1 2		2054-5	Black or African American		362,803 362,803	11.4% 11.4%		1	<0.001

Appendix C 

**Table 5:** Patient number and demographic characteristics in cohort 1 (influenza vaccine) and cohort 2 (mRNA vaccine) before and after propensity score matching.

Cohort 1 and cohort 2 patient count before and after propensity score matching											
			Cohort			Patient count before matching			Patient count after matching		
			1 - Influenza vaccine			2,355,057			2,190,260		
			2 - mRNA vaccine			3,177,285			2,190,260		
Cohort 1 (N = 2,355,057) and cohort 2 (N = 3,177,285) characteristics before propensity score matching											
	Demographics										
		Cohort				Mean ± SD	Patients	% of Cohort		P-Value	Std diff.
		1 2		AI	Age at Index	47.2 +/- 21.7 52.2 +/- 19.1	2,355,057 3,177,285	100% 100%		<0.001	0.242
		1 2		2106-3	White		1,658,878 2,011,825	70.4% 63.3%		<0.001	0.152
		1 2		F	Female		1,396,539 1,801,876	59.3% 56.7%		<0.001	0.052
		1 2		2054-5	Black or African American		313,189 362,803	13.3% 11.4%		<0.001	0.057
Cohort 1 (N = 2,190,260) and cohort 2 (N = 2,190,260) characteristics after propensity score matching											
	Demographics										
		Cohort				Mean ± SD	Patients	% of Cohort		P-Value	Std diff.
		1 2		AI	Age at Index	49.7 +/- 20.4 51.0 +/- 19.4	2,190,260 2,190,260	100% 100%		<0.001	0.066
		1 2		2106-3	White		1,530,654 1,581,403	69.9% 72.2%		<0.001	0.051
		1 2		F	Female		1,276,010 1,305,657	58.3% 59.6%		<0.001	0.028
		1 2		2054-5	Black or African American		276,616 281,668	12.6% 12.9%		<0.001	0.007

Appendix D 

**Table 6:** Patient number and demographic characteristics in cohort 1 (Tdap/Td) and cohort 2 (mRNA vaccine) before and after propensity score matching. Tdap/Td denotes tetanus, diphtheria, and pertussis/tetanus and diphtheria.

Cohort 1 and cohort 2 patient count before and after propensity score matching											
			Cohort			Patient count before matching			Patient count after matching		
			1 - Tdap/Td vaccines			1,817,211			1,630,042		
			2 - mRNA vaccine			3,177,285			1,630,042		
Cohort 1 (N = 1,817,211) and cohort 2 (N = 3,177,285) characteristics before propensity score matching											
	Demographics										
		Cohort				Mean ± SD	Patients	% of Cohort		P-Value	Std diff.
		1 2		AI	Age at Index	41.4 +/- 18.9 52.2 +/- 19.1	1,817,211 3,177,285	100% 100%		<0.001	0.568
		1 2		2106-3	White		1,220,548 2,011,825	67.2% 63.3%		<0.001	0.081
		1 2		F	Female		1,025,614 1,801,876	56.4% 56.7%		<0.001	0.005
		1 2		2054-5	Black or African American		269,601 362,803	14.8% 11.4%		<0.001	0.101
Cohort 1 (N = 1,630,042) and cohort 2 (N = 1,630,042) characteristics after propensity score matching											
	Demographics										
		Cohort				Mean ± SD	Patients	% of Cohort		P-Value	Std diff.
		1 2		AI	Age at Index	44.1 +/- 17.9 44.4 +/- 17.5	1,630,042 1,630,042	100% 100%		<0.001	0.018
		1 2		2106-3	White		1,089,009 1,125,192	66.8% 69.0%		<0.001	0.048
		1 2		F	Female		927,051 908,333	56.9% 55.7%		<0.001	0.023
		1 2		2054-5	Black or African American		220,635 209,662	13.5% 12.9%		<0.001	0.020
